# Interaction between microRNA expression and classical risk factors in the risk of coronary heart disease

**DOI:** 10.1038/srep14925

**Published:** 2015-10-08

**Authors:** Xiao-Qing Ding, Peng-Cheng Ge, Zhe Liu, Heng Jia, Xi Chen, Feng-Hui An, Li-Hua Li, Zhao-Hong Chen, Hong-Wei Mao, Zhao-Yang Li, Yan Gu, Tie-Bing Zhu, Chun-Jian Li, Lian-Sheng Wang, Wen-Zhu Ma, Zhi-Jian Yang, En-Zhi Jia

**Affiliations:** 1 First Affiliated Hospital of Nanjing Medical University, Nanjing 210029, Jiangsu Province, China; 2 Kangda school, Nanjing Medical University, Lianyungang 222000, Jiangsu Province, China; 3 Jiangsu Engineering Research Center for MicroRNA Biology and Biotechnology, State Key Laboratory of Pharmaceutical Biotechnology, School of Life Sciences, Nanjing University, Nanjing 210029, Jiangsu Province, China; 4 Friendship Hospital of Ili Kazakh Autonomous Prefecture, Yining 835000, Xinjiang, China

## Abstract

The aim of this study was to identify the synergistic effect of microRNA expression with classical risk factors of coronary heart disease (CHD) and to explore their diagnostic value for coronary stenotic lesions in subjects with CHD. Plasma samples were obtained from 66 subjects with CHD and from 58 control individuals. A quantitative reverse-transcription PCR (RT-qPCR) assay was conducted to confirm the relative expressions of the known CHD-related miRNAs. The severity of coronary atherosclerosis was based on the Gensini scoring system. The expression of miR-125b in plasma of the CHD group was lower than that of the non-CHD group (0.14 ± 0.09 *vs*. 0.18 ± 0.10, *p*** = **0.055), and the miR-125b levels significantly decreased following an increasing Gensini score (P** = **0.037). Spearman correlation analyses indicated the Gensini score was negatively associated with miR-125b (*r*** = **−0.215, *p*** = **0.017). Of all the miRNAs, miR-125b showed the lowest *AUC* (0.405; 95% *CI*: 0.305 ~ 0.506, *p*** = **0.070). We found several synergistic effects between miR-125b and classical risk factors, such as age, sex, CR, FBG and HDL-C; the proportion of CHD attributable to the interaction of miR-125b and age was as high as 80%. Therefore, miR-125b was shown to play an important role in individual’s susceptibility to developing CHD.

Cardiovascular diseases (CVDs) are the leading causes of morbidity and mortality worldwide. Over the past few decades, the diagnosis, treatment, and prognosis of coronary heart disease (CHD) has dramatically changed and has led to a decrease in the mortality risk. However, there is still a clinical need for new diagnostic and prognostic biomarkers and novel modern interventions to reduce the incidence of CHD^1^. Recently, microRNAs (miRNAs) have been shown to be associated with CHD^2^,^3^,^4^. MiRNAs are small noncoding RNA molecules that negatively regulate gene expression by triggering either translation repression or RNA degradation on the post-transcriptional level^5^,^6^. Approximately 1000 miRNAs have been found in humans to date. Bioinformatics and cloning studies have estimated that miRNAs regulate 30% of all human genes^7^,^8^. Among them, there are approximately 50 circulating miRNAs believed to be associated with cardiovascular diseases^1^. By analysing the profiles of miRNAs released into the blood plasma or serum from cells and tissues of the cardiovascular system in CHD patients, it has been shown that some miRNAs are down-regulated while others are up-regulated^9^. Many studies have demonstrated the role of cardiomyocyte-enriched miRNAs (miR-1, miR133a, miR-133b, miR-208a, miR-208b, and miR-499) in cardiac damage and myocardial infarction; moreover, some studies have highlighted the underlying effect of circulating miRNAs as diagnostic and prognostic biomarkers in AMI^10^.

Although coronary angiography has significantly improved the early diagnosis of CHD, it remains a major clinical challenge. Genetic epidemiology is increasingly focused on the study of common diseases with both genetic and environmental determinants. The concept of a gene-environment interaction is becoming a central theme in epidemiologic studies that assess the causes of human disease in populations^11^. However, the microRNA-environment interaction on CHD has not yet been reported. Therefore, we identified the synergistic effect of microRNA expression with classical risk factors on CHD and explored their respective diagnostic value for coronary stenotic lesions in subjects with CHD.

## Subject and Methods

### Study subjects

From 2012 to 2013, 124 consecutive subjects (91 males and 33 females), aged 37–77 years, who underwent coronary angiography for suspected or known coronary atherosclerosis at the Friendship Hospital of Ili Kazakh Autonomous Prefecture in China were enrolled in this study. Among them, 66 subjects with CHD (54 males and 12 females, age 39–77 years) and 58 subjects (37 males and 21 females, age 37–73 years) with angiographic exclusion of CHD served as the control group. General exclusion criteria included the following: subjects with spastic angina pectoris, infectious processes within 2 weeks, heart failure, adrenal dysfunction, and thyroid dysfunction. The methods were performed in accordance with the approved guidelines, and all experimental protocols were approved by the ethics committee of the First Affiliated Hospital of Nanjing Medical University and the Friendship Hospital of Ili Kazakh Autonomous Prefecture in China. All subjects provided written informed consent.

### Coronary Angiography

Coronary arteries were cannulated using either the Judkins technique^12^ or through a radial artery approach with 6F catheters and recorded at a rate of 30 frames/s. The presence of coronary artery stenosis was evaluated after the direct intracoronary injection of isosorbide dinitrate (ISDN; 2.5 mg/5 ml solution over 20 s). One minute after the injection of ISDN through the Judkins catheter, coronary angiography was performed from several projections. Coronary angiograms were evaluated independently by operators who made visual estimations of the luminal narrowing in multiple segments, based on the AHA/ACC classification, of the coronary tree. Significant CHD was defined as at least one major epicardial vessel with >50% stenosis; the control was defined as all of the major epicardial vessels with <50% stenosis^13^. The severity of the coronary atherosclerosis was based on the Gensini scoring system. The Gensini score was computed by assigning a severity score in each coronary stenosis according to the degree of luminal narrowing and its geographic importance. Reduction in the lumen diameter and the roentgenographic appearance of concentric lesions and eccentric plaques were evaluated (reductions of 25%, 50%, 75%, 90%, 99% and complete occlusion were given Gensini scores of 1, 2, 4, 8, 16 and 32, respectively). Each principal vascular segment was assigned a multiplier according to the functional significance of the myocardial area supplied by that segment: the left main coronary artery, ×5; the proximal segment of left anterior descending coronary artery (LAD), ×2.5; the proximal segment of the circumflex artery, ×2.5; the mid-segment of the LAD, ×1.5; the right coronary artery, the distal segment of the LAD, the posterolateral artery, and the obtuse marginal artery, ×1; and all others, ×0.5^14^.

### Laboratory measurements

Four millilitres of venous blood was drawn after 12 hours of fasting to perform biochemical assays in the routine laboratory on the second day of hospitalization; the total cholesterol (TCH, mmol/L), triglyceride (TG, mmol/L), fasting blood glucose (FBG, mmol/L), creatinine (CR, umol/L), fasting high-density lipoprotein cholesterol (HDL-C, mmol/L) and fasting low-density lipoprotein cholesterol (LDL-C, mmol/L) were determined by enzymatic procedures on an automated autoanalyzer (AU 2700 Olympus, 1st Chemical Ltd, Japan). Excellent intra-assay and inter-assay CVs of <5% were obtained with our assay method.

### Selection of miRNAs

Based on previous studies, 20 CHD-related miRNAs were employed as candidates in the study: miR-433, miR-485-3p, miR-1, miR-122, miR-133a, miR-133b, miR-145^15^, miR-214^16^, miR-21, miR-25, miR-20a, miR-106a, miR-92a^17^, miR-130a, miR-155, miR-221, miR-208a, miR-208b, miR-499^18^, and miR-125b^19^,^20^,^21^,^22^.

### Plasma preparation and RNA isolation

All samples (5 ml) were collected in EDTA plasma tubes on the morning following arrival. Samples were processed within 4 hours and stored at 4 °C. Plasma was collected after centrifugation (15 min at 1000 × g) and was stored at −80 °C until further analysis was performed.

For the RT-qPCR assay of plasma, total RNA was extracted using a one-step phenol/chloroform purification protocol, as Cheng Wang *et al*. reported^23^ in 2011. Briefly, 100 μl of plasma was mixed with 300 μl of RNase-free water, 200 μl of phenol and 200 μl of chloroform. After 10 min at room temperature, the mixture was centrifuged at 16,000 × *g* for 20 min. After phase separation, the upper aqueous layer was mixed with 2 volumes of isopropyl alcohol and 0.1 volumes of 3 M sodium acetate (pH** = **5.3). The total RNA was precipitated at −20 °C for 1 hour. The RNA pellet was collected by centrifugation at 16,000 × g for 20 min. The pellet was subsequently washed with 75% ethyl alcohol and dried for 10 min at room temperature. Finally, the RNA was dissolved in 20 ml of RNase-free water and stored at −80 °C until further analysis was performed.

### Quantification of miRNAs by RT-qPCR analysis

For miRNA profiling, the RT-qPCR assay was performed using a TaqMan PCR kit according to the manufacturer’s instructions (Applied Biosystems, Foster City, USA); a minor modification was made according to the State Key Laboratory of Pharmaceutical Biotechnology (School of Life Sciences, Nanjing University), reported in 2010^24^. TaqMan miRNA assays are all available through Applied Biosystems. In brief, the reverse transcription reaction was performed with a final volume of 10 μl, which included 2 μl of plasma extract RNA, 0.5 μl of AMV reverse transcriptase (TaKaRa), 1 μl of stem-loop RT primer (Applied Biosystems), 1 μl of 10 mMdNTPs, 2 μl of 5× reverse transcription buffer and 3.5 μl of RNase-free water. For synthesis of cDNA, the reaction solutions were incubated at 16 °C for 30 min, at 42 °C for 30 min, at 85 °C for 5 min, and then held at 4 °C. One reverse transcriptase reaction with no-template control was included. The quantitative PCR reaction was then performed in 20 μl, containing 1 μl of cDNA, 0.3 μl of Taq polymerase, 0.33 μl of TaqMan probe, 0.4 μl of 10 mMdNTPs, 1.2 μl of 25 mM MgCl_2_, 2 μl of 10 × PCR buffer (MgCl_2_ free) and 14.77 μl of RNase-free water. Real-time PCR was performed with the Roche Molecular Systems Mastercycler® ep realplex with one cycle of 95 °C for 5 min, followed by 40 cycles of 95 °C for 15 sec and 60 °C for 60 sec. All reactions were run in triplicate, containing three wells of product of reverse transcriptase reaction with no-template control and water blanks as negative controls. Due to the superior performance of a combination of let-7d, let-7g and let-7i, this combination was chosen as a reference for the normalization of plasma miRNAs rather than the commonly used reference genes U6, RNU44, RNU48 and miR-16^25^. The resulting threshold cycle (CT) values were determined according to the default threshold settings when the reactions were completed. The relative amount of each miRNA was calculated based on the internal control, i.e., the combination of let-7d, let-7g and let-7i, analysed using the 2^−Δct^ method, which is a widely used method for presenting relative gene expression by comparative CT, and the calculation formula was as following: 2exp-(mean Ct target miRNA-mean Ct internal control)^26^,^27^. Present the data as 2^−Δct^ and plot the heatmap as described by means of *MultiExperiment Viewer v4.9*^28^.

### Statistical analysis

Data were statistically analysed using Statistics Package for Social Sciences (ver. 16.0; SPSS Incorporated, Chicago, IL, USA). Subjects were classified into 2 groups according to CHD status and 4 groups according to the quartile of the Gensini score. Data for age, TCH, CR, HDL-C, LDL-C, miR-20a, and miR-125b were normally distributed parameters and presented as the mean ± SD; comparisons were performed using the independent-samples T test and one-way ANOVA. Skewed data, including the TG, FBG, Gensini scores, miR-485-3p, miR-133b, miR-221, miR-208a, miR-433, miR-1, miR-122, miR-133a, miR-145, miR-214, miR-21, miR-25, miR-106a, miR-92a, miR-130a, miR-155, miR-208b, and miR-499 were expressed as median and quartile ranges, and comparisons were performed using the Mann-Whitney U test and Kruskal-Wallis H test. Categorical variables of gender were compared between the groups of patients using a chi-squared analysis. The Spearman two-way test was used to assess the relationship between Gensini scores with miRNAs and classical risk factors. As a measure of effect strength and direction when using the Mann–Whitney test, the area under the receiver operating characteristic (*ROC*) curve was computed^29^.

To analyse possible positive or negative interactions between microRNA expression with classical risk factors, the 4 × 2 table approach was used to calculate odds ratio (*OR*), respective 95% confidence intervals (*CI*) and two-tailed *p* values; additionally, this approach was used in synergy measures in additive (*SI*) or multiplicative models (*SIM*). It was assumed that unexposed individuals without the susceptibility microRNA had a certain background risk for CHD (*OR*00 is assumed to be 1); *OR*10 refers to the relative risk for CHD among people without the susceptibility microRNA for CHD but exposed to the environmental risk factor relative to those with neither the susceptibility microRNA nor exposure; *OR*01 refers to the relative risk among people with the susceptibility microRNA who are not exposed to the risk factor relative to those with neither the susceptibility microRNA nor exposure; and *OR*11 is the ratio of CHD risk among exposed people with susceptibility microRNA to CHD risk among unexposed people without the susceptibility microRNA. These *OR*s were then used in the calculation of synergy indices: *SI* **= ** (*OR*11 − 1)/(*OR*10 + *OR*01 − 2), *SIM*** = ***OR*11/(*OR*10 × *OR*01); the relative excess risk due to interaction, *RERI*** = ***OR*11 − *OR*10 − *OR*01 + 1; and the attributable proportion of the disease due to interaction, *AP*** = ***RERI*/*OR*11^11^,^30^. The susceptibility microRNAs, environment risk factors and their cut-offs were determined using the area under the receiver operating characteristic (*ROC*) curve.

Differences were considered to be significant if the null hypothesis could be rejected with >95% confidence. All *p*-values were two-tailed.

## Results

### Characteristics of the study population grouped by CHD and controls

Table 1 presents the characteristics of the study population. A total of 66 subjects with CHD and 58 controls were enrolled in the study. As expected, increasing age (*p*** = **0.000) and male (*p*** = **0.023) are risk factors for CHD in this population. Additionally, the level of HDL-C (*p*** = **0.020), FBG (*p*** = **0.006), CR (*p*** = **0.033), and Gensini scores (*p*** = **0.000) were significantly different between CHD and controls.

**Figure 1:**
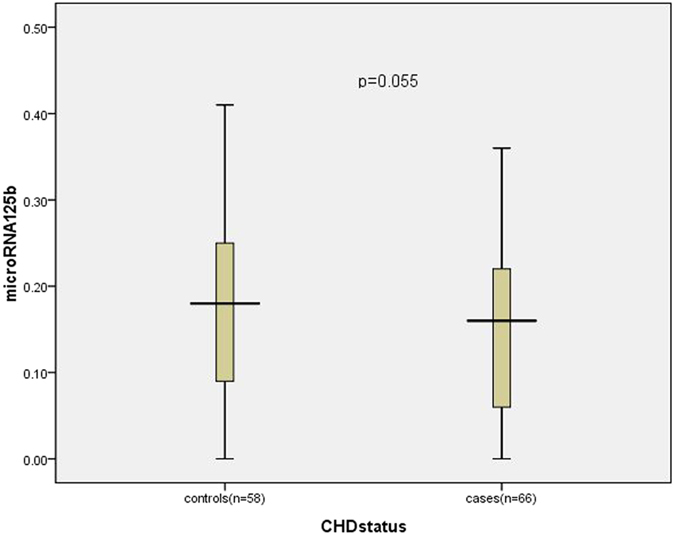
The relative expression of miR-125b in plasma between controls and CHD cases.

To determine miRNAs in CHD, we compared the levels of 20 circulating miRNAs in plasma samples of CHD subjects to controls. The relative expression of miR-125b in plasma of the CHD group (n** = **66) was 0.14 ± 0.09, lower than that of the non-CHD group (n** = **58), which was 0.18 ± 0.10 (*p*** = **0.055) (Fig. 1); and the relative expression of miR-133b in plasma of the CHD group lower than that of the non-CHD group (0.14(0.06 ~ 0.23) *vs* 0.17(0.11 ~ 0.22), *p*** = **0.207); however, no significant difference was reached.

### Characteristics of the study population categorized by the quartile of Gensini score

**Figure 2:**
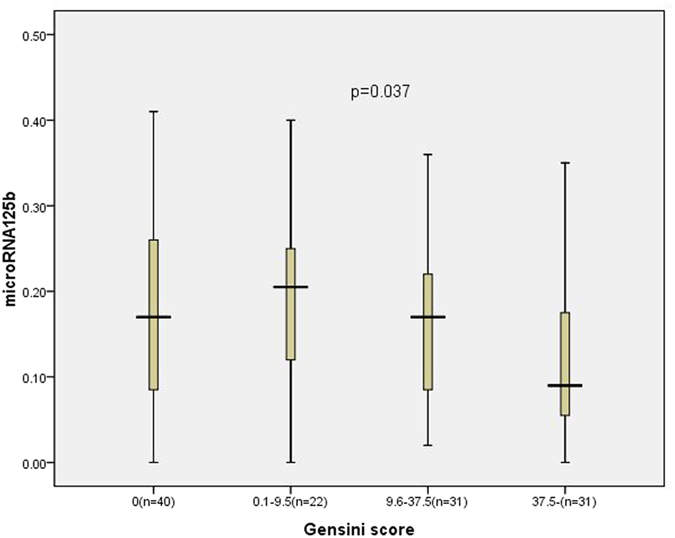
The relative expression of miR-125b in plasma among the subjects categorized by the quartile of the Gensini score.

Subjects were divided into 4 groups according to the quartiles of the Gensini score: 0 (first quartile; n** = **40 subjects), 0.1 to 9.5 (second quartile; n** = **22 subjects), 9.6 to 37.5 (third quartile; n** = **31 subjects) and ≥37.5 (fourth quartile; n** = **31 subjects). The characteristics of the study population categorized by the quartile of the Gensini score are shown in Table 2. Age (*P*** = **0.001) and serum CR levels (*P*** = **0.003) significantly increased as the involvement of the Gensini score increased. The prevalence of CHD (*P*** = **0.000) and the proportion of male gender (*P*** = **0.009) were also significantly greater in the highest quartile of the Gensini score. Moreover, plasma miR-125b levels significantly decreased following an increasing Gensini score (*P*** = **0.037) (Fig. 2).

### Spearman correlations between Gensini score with clinical characteristics and miRNAs

**Figure 3:**
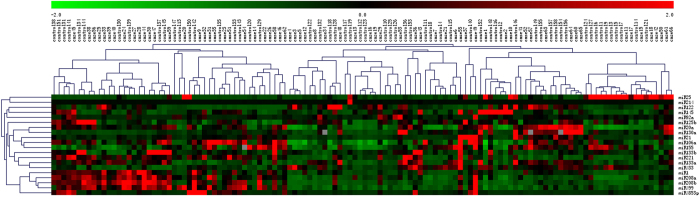
The heatmap of real-time PCR expression profiling of microRNA in subjects with CHD and controls.

Table 3 shows the results of Spearman correlation analyses between Gensini score, clinical characteristics, and miRNAs. The results indicate that the Gensini score was positively associated with age (*r*** = **0.339, *p*** = **0.000), FBG (*r*** = **0.223, *p*** = **0.014), and CR (*r*** = **0.276, *p*** = **0.002). A negative association was found between the Gensini score and HDL-C (*r*** = **−0.211, *p*** = **0.021), and miR-125b (*r*** = **−0.215, *p*** = **0.017). And, the heatmap was generated by transformation of the real-time PCR data presented as 2^−Δct^ and was shown in Fig. 3.

### Receiver operating characteristic curve analyses in subjects with CHD and controls

**Figure 4:**
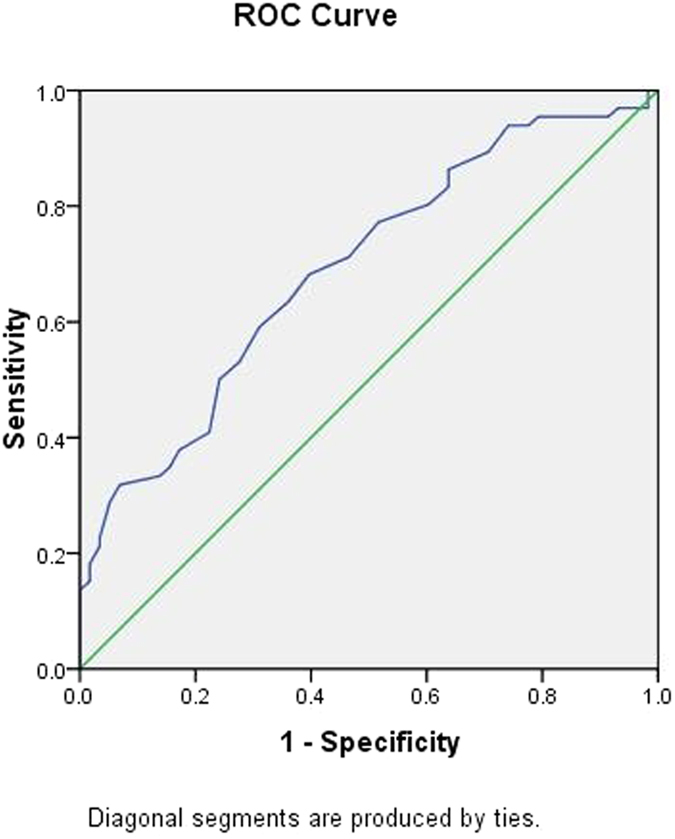
The receiver operating characteristic curve of age for the ability to differentiate the CHD cases from the control individuals, *AUC* (95% *CI*) was 0.695(0.601 ~ 0.788).

**Figure 5:**
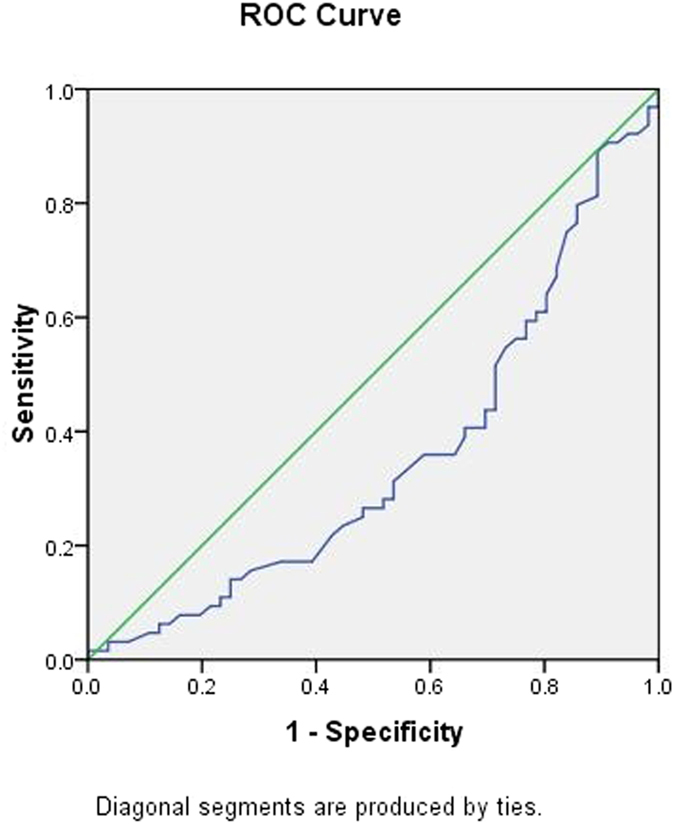
The receiver operating characteristic curve of HDL-C for the ability to differentiate the CHD cases from the control individuals, *AUC* (95% *CI*) was 0.354(0.255 ~ 0.454).

**Figure 6:**
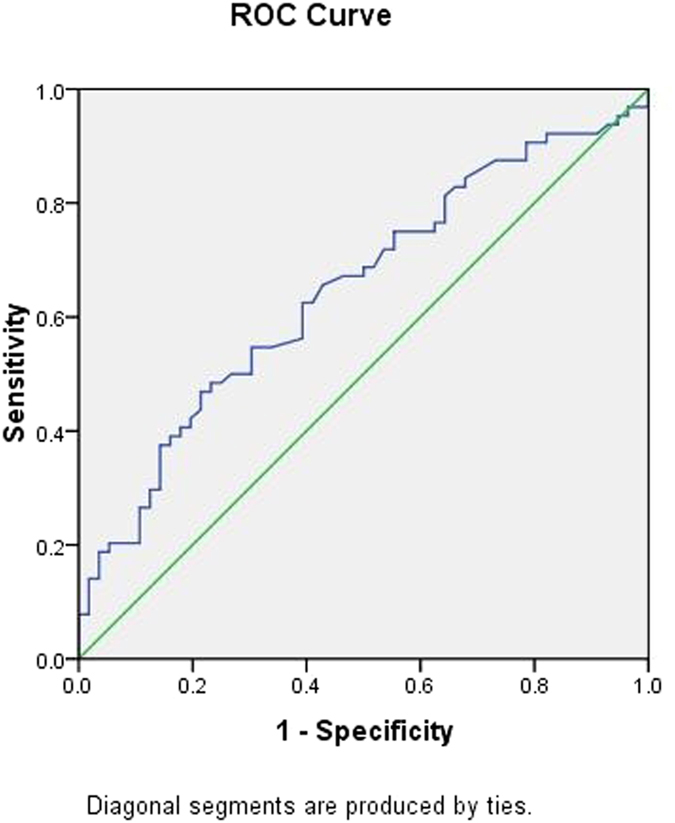
The receiver operating characteristic curve of FBG for the ability to differentiate the CHD cases from the control individuals, *AUC* (95% *CI*) was 0.647(0.549 ~ 0.745).

**Figure 7:**
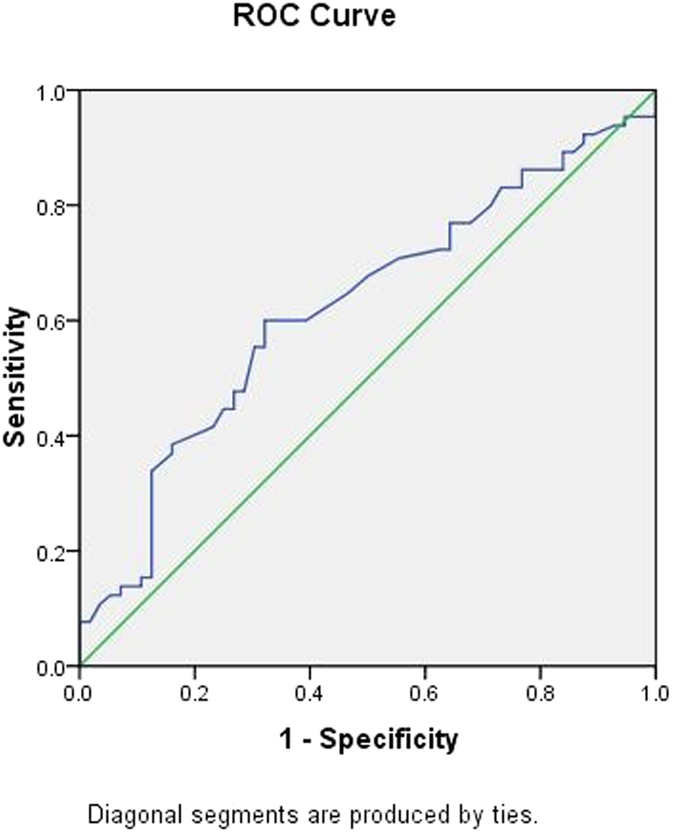
The receiver operating characteristic curve of CR for the ability to differentiate the CHD cases from the control individuals, *AUC* (95% *CI*) was 0.622(0.522 ~ 0.723).

**Figure 8:**
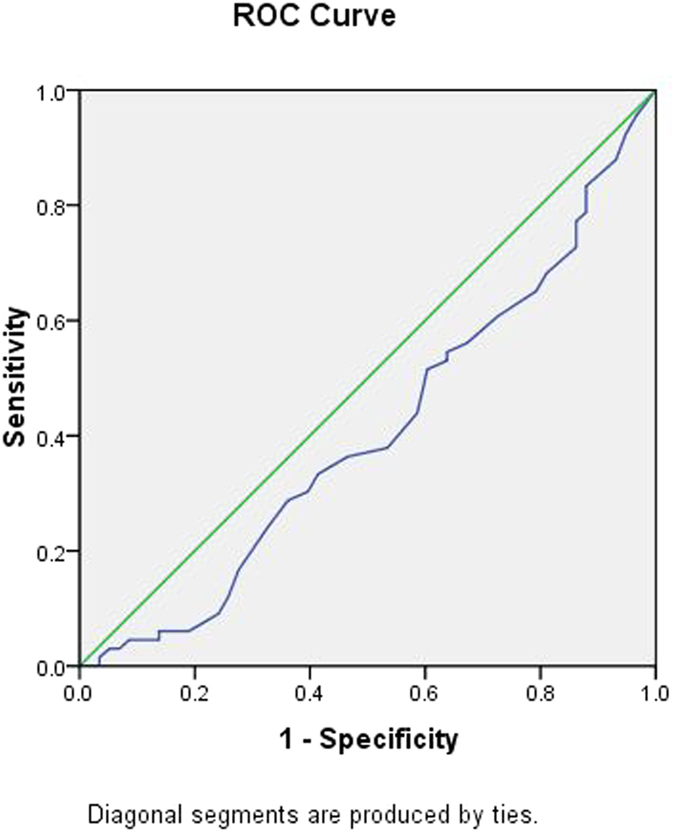
The receiver operating characteristic curve of miR-125b for the ability to differentiate the CHD cases from the control individuals, *AUC* (95% *CI*) was 0.405(0.305 ~ 0.506).

To further explore the applicability of circulating miRNAs and classical risk factors as potential diagnostic biomarkers of CHD, subsequent *ROC* analyses were performed, and the results are shown in Table 4. The *AUC* was 0.695 for age (95% *CI*: 0.601 ~ 0.788, *p*** = **0.000) (Fig. 4); 0.354 for HDL-C (95% *CI*: 0.255 ~ 0.454, *p*** = **0.006) (Fig. 5); 0.647 for FBG (95% *CI*: 0.549 ~ 0.745, *p*** = **0.006) (Fig. 6); and 0.622 for CR (95% *CI*: 0.522 ~ 0.723, *p*** = **0.021) (Fig. 7). Of all the miRNAs, miR-125b showed the lowest *AUC* (0.405; 95% CI: 0.305 ~ 0.506, *p*** = **0.070) (Fig. 8). Due to the levels of HDL-C, miR-133b and miR-125b were lower in patients with CHD than in the controls; the *AUC* of the above three variables were less than 0.5. The optimal cut-off value, the sensitivity, the specificity and Youden index of age, CR, FBG, HDL-C, and miR-125b are shown in Table 5.

### Interaction between miR-125b and classical risk factors

The results from the analysis of the possible positive/negative association between miR-125b and classical risk factors are presented in Tables 6 and 7. According to the *ROC* results, the cut-off of 0.175 for expression of miR-125b was the optimal value, accordingly, subjects with expression of miR-125b less than 0.175 were employed as subjects with susceptibility microRNA. Regarding the conventional risk of subjects unexposed to both classical risk factor and miR-125b risk (reference category) as being 1.0, the *ORs* estimating the effect of joint exposure to miR-125b and age, sex (male was defined as risk gender), CR, FBG, and HDL-C were significantly higher than the *OR*s estimating the effect of each factor in the absence of the other. A negative association between miR-125b and age was found (*SI* **= **−50.99, *SIM* **= **5.48, *AP*** = **0.80), the proportion of CHD attributable to the interaction of miR-125b and age in this group was as high as 80%. After performing the same analysis regarding gender, significant results was found in male subjects with susceptibility microRNA of miR-125b (*SI* **= **1.18, *SIM* **= **0.78, *AP*** = **0.12). The susceptibility microRNA of miR-125b (*SI* **= **1.46, *SIM* **= **1.00, *AP*** = **0.25) interact with CR to develop CHD. The risk provided by FBG was found to be positively reinforced by susceptibility microRNA of miR-125b (*SI*** = **1.25, *SIM*** = **0.77, *AP*** = **0.16) in this study. The HDL-C and miR-125b significantly interact during the onset of CHD; this combination was shown to be accountable for 28% of the disease (*SI*** = **1.50, *SIM*** = **0.92, *AP*** = **0.28).

## Discussion

To the best of our knowledge, this is the first study to explore the interaction between microRNA expressions with classical risk factors on coronary heart disease throughout the world. Based on the data of 66 subjects with CHD and 58 subjects with angiographic exclusion of CHD, interactions between miR-125b and classical risk factors were found; furthermore, the proportion of CHD attributable to the interaction of miR-125b and age was as high as 80%.

Cardiovascular diseases are the major cause of death worldwide, particularly in the elderly population who have an increasing rate of mortality and morbidity; cardiovascular diseases are a consequence of genetic and epigenetic interactions^31^,^32^,^33^. The most important epigenetic modifications of mammalian cells are associated with DNA methylation, posttranslational histone modifications, and a class of short noncoding RNAs, the microRNAs (miRNAs or miRs)^34^,^35^. MicroRNAs are endogenous, small, non-coding RNAs involved in the regulation of gene expression^36^. They control expression on a posttranscriptional level as intracellular RNAs and have been discussed as potential therapeutic targets^37^. MicroRNAs are involved in the pathogenesis of various cardiovascular conditions, such as CHD. We hypothesize that CHD has a multifactorial genetic basis involving a number of microRNAs and interacting environmental factors to determine the potential development of disease. The present study has proven this hypothesis. Therefore, the expression of microRNAs generally predisposes to a greater or lesser extent of CHD, but it is the environmental factors interacting with the individual's expression of microRNAs that determine whether CHD will develop.

The muscle-enriched miRNA, miR-133, is almost the most abundant of the miRNAs present in the normal heart^38^,^39^; the gene MIR133b encodes miR-133b. There is evidence that muscle- and/or cardiac-specific miRNA-133b is involved in heart development and some cardiovascular diseases, including myocardial infarction^40^,^41^, myocardial injury after operation^42^,^43^ and cardiomyopathy^44^,^45^. In the present study, the circulating miRNA expression level of miR-133b was lower in subjects with CHD than those in controls, these observations are in line with the results of recently published studies^40^,^41^,^42^,^43^, showing lower miR-133b levels in patients with CHD than in controls, however, no significant difference was reached regarding as the relative expression of miR-133b between the cases and controls in the present study.

Mature miR-125b originates from two precursors: pre-miR-125b-1 (located at chr11q24.1) and pre-miRNA-125b-2 (located at chr21q21.1)^46^. A number of studies have investigated the relationships between the members of the miR-125 family, particularly miR-125b, and various diseases, including solid tumours^47^,^48^,^49^, hepatological malignancies^50^,^51^, autoimmune diseases^52^,^53^, lung disease^54^,^55^, chronic kidney disease^56^, acute stroke^57^ and obesity^58^. However, there is little data to support the involvement of miR-125b in human CHD. Suli Huang *et al*.^59^ reported that the plasma levels of miR-320b and miR-125b were significantly lower in patients with AMI than in controls. Because miR-320b and miR-125b were expressed in vascular endothelial cells (VECs), the authors hypothesized that the down-regulated miRNAs in AMI might be related to the development and progression of atherosclerosis; this may then trigger myocardial infarction by regulating gene expressions in VECs. The preliminary functional study^60^ suggested that miR-125a-5p and miR-125b-5p were highly expressed in VECs and that both miR-125a/b-5p could suppress oxLDL-induced endothelin-1 (ET-1) expression by directly targeting the 3′untranslated region of preproET-1 mRNA. Accumulating evidence has suggested that ET-1 plays a significant role in many physiologic and pathophysiological processes, including heart development^61^, cardiac hypertrophy^62^, and atherosclerosis^63^. These functional analyses could further the understanding of the role of circulating miRNAs in atherosclerosis processes and pave the way for further studies. Of all the miRNAs in the present study, miR-125b showed the lowest *AUC* (0.405; 95% *CI*: 0.305 ~ 0.506, *p*** = **0.070). To analyse possible positive or negative interactions between miR-125b expressions with classical risk factors, a 4 × 2 table approach was used; the synergy measures in additive (*SI*) or multiplicative models (*SIM*) of miR-125b with age, male gender, CR, FBG, and HDL-C were −50.99 or 5.48, 1.18 or 0.78, 1.46 or 1.00, 1.25 or 0.77, and 1.50 or 0.92, respectively. Therefore, the positive interactions between miR-125b with male gender, CR, FBG, and HDL-C were found, and the proportion of CAD attributable to the interaction between HDL-C and susceptibility miR-125b was approximately 28% for the study. Nevertheless, we found a negative interaction between miR-125b expressions with age; the proportion of CHD attributable to the interaction of age and miR-125b was as high as 80%.

In summary, the present study provides the first insights into the interaction between microRNA expressions with classical risk factors of coronary heart disease around the world. Based on the data of 66 subjects with CHD and 58 control individuals, interactions between miR-125b and classical risk factors were found; the proportion of CHD attributable to the interaction of miR-125b and age was as high as 80%. The results of the present study may have important implications for better understanding of the aetiology and pathogenesis of the disease through quantitative assessment of disease risks in some populations. Considering that CHD is a multifactorial and multigenic disease driven by various environmental and genetic components interacting together, our results appear to support the hypothesis that the susceptibility microRNA may indeed enhance the influence of classical risk factors in the development of CHD. However, this was a single-centre study conducted in China, and the conclusions drawn based on this study’s results may not be applicable to other populations. Furthermore, the mechanisms underlying the dysregulation remain to be determined. Therefore, further prospective large-scale studies are required to determine the potential role of circulating miRNAs in the microRNA-environment, interaction, and diagnostic value of coronary stenotic lesions in subjects with CHD.

## Additional Information

**How to cite this article**: Ding, X.-Q. *et al*. Interaction between microRNA expression and classical risk factors in the risk of coronary heart disease. *Sci. Rep*. **5**, 14925; doi: 10.1038/srep14925 (2015).

## Figures and Tables

**Table 1:** Characteristics of the study population categorized by CHDs and controls.

Characteristics	CHDs (N = 66)	Controls (N = 58)	Statistical parameter	*P*value
Age (years)	62.20 ± 8.95	55.93 ± 8.68	−3.946	0.000
Gender (F/M)	12/54	21/37	5.136	0.023
TCH	4.75 ± 1.04	4.64 ± 0.99	−0.606	0.546
HDL-C	1.35 ± 0.33	1.50 ± 0.34	2.354	0.020
LDL-C	2.93 ± 0.91	2.76 ± 0.85	−1.045	0.298
FBG	5.44(4.88 ~ 6.34)	5.04(4.71 ~ 5.50)	1266.00	0.006
CR	74.10 ± 15.87	68.56 ± 11.62	−2.161	0.033
Gensini scores	35.50(16.75 ~ 64.50)	0.00(0.00 ~ 2.00)	51.000	0.000
miR-433	0.60(0.31 ~ 1.15)	0.62(0.37 ~ 1.13)	1868.500	0.820
miR-1	1.56(0.49 ~ 3.18)	0.87(0.44 ~ 2.18)	1651.500	0.189
miR-122	0.48(0.12 ~ 1.42)	0.31(0.10 ~ 0.8)	1733.000	0.366
miR-133a	0.43(0.19 ~ 0.9)	0.35(0.23 ~ 0.79)	1810.000	0.602
miR-145	0.10(0.04 ~ 0.17)	0.08(0.04 ~ 0.18)	1829.000	0.670
miR-214	0.18(0.04 ~ 1)	0.13(0.03 ~ 0.49)	1698.500	0.280
miR-21	0.61(0.38 ~ 1.01)	0.64(0.42 ~ 1.09)	1763.500	0.451
miR-25	0.02(0.01 ~ 0.08)	0.02(0.01 ~ 0.08)	1851.000	0.747
miR-20a	0.38 ± 0.26	0.39 ± 0.33	0.028	0.978
miR-106a	2.41(1.51 ~ 3.99)	2.69(1.79 ~ 4.31)	1757.000	0.432
miR-92a	0.19(0.04 ~ 0.78)	0.20(0.08 ~ 1.16)	1707.500	0.301
miR-130a	54.35(29.32 ~ 111.82)	61.82(35.26 ~ 107.49)	1880.500	0.867
miR-155	197.19(102.36 ~ 371.59)	201.56(118.86 ~ 322.18)	1908.500	0.978
miR-208b	1.13(0.63 ~ 4.13)	1.02(0.6 ~ 3.56)	1791.500	0.540b
miR-499	3.5(2.19 ~ 8.62)	3.09(2.03 ~ 6.66)	1739.500	0.382
miR-485-3p	1.79(0.97 ~ 5.53)	1.54(0.76 ~ 3.35)	1708.000	0.302
miR-133b	0.14(0.06 ~ 0.23)	0.17(0.11 ~ 0.22)	1662.000	0.207
miR-221	0.4(0.19 ~ 0.82)	0.55(0.22 ~ 0.92)	1740.000	0.384
miR-208a	1.13(0.63 ~ 4.13)	1.87(1.19 ~ 3.78)	1632.500	0.159
miR-125b	0.14 ± 0.09	0.18 ± 0.10	1.938	0.055

Data are summarized by either mean ± standard deviation or 50th (25th/75th) percentiles for continuous variables and N_1_/N_2_ for binary variables. CHD, coronary heart disease; TCH, total cholesterol; HDL-C, fasting high-density lipoprotein cholesterol; LDL-C, fasting low-density lipoprotein cholesterol; TG, triglyceride; FBG, fasting blood glucose; CR, creatinine. The value of each miRNAs means the relative mount calculated by 2^−Δct^ method.

**Table 2:** Characteristics of the study population categorized by the quartile of Gensini score.

Characteristics	Gensini score				Statistical parameter	*P*value
	0(n = 40)	0.1–9.5(n = 22)	9.6–37.5(n = 31)	37.5-(n = 31)		
Age (years)	55.70 ± 8.41	56.86 ± 9.50	61.68 ± 9.14	63.16 ± 8.70	5.460	0.001
Gender (F/M)	13/27	11/11	5/26	4/27	11.599	0.009
TCH	4.72 ± 0.97	4.52 ± 1.07	4.79 ± 1.10	4.72 ± 0.97	0.313	0.816
HDL-C	1.48 ± 0.35	1.51 ± 0.33	1.33 ± 0.28	1.37 ± 0.39	1.846	0.143
LDL-C	2.82 ± 0.83	2.61 ± 0.86	3.01 ± 1.00	2.89 ± 0.85	0.936	0.426
FBG	5.07(4.64–5.73)	5.04(4.80–5.49)	5.08(4.78–6.29)	5.89(4.95–6.99)	7.610	0.055
CR	69.73 ± 12.62	63.11 ± 7.85	74.73 ± 19.35	76.71 ± 10.58	5.048	0.003
CHD (case/control)	0/40	5/17	30/1	31/0	104.600	0.000
miR-433	0.57(0.32 ~ 1.09)	0.77(0.47 ~ 1.38)	0.57(0.31 ~ 1.14)	0.58(0.28 ~ 1.04)	2.959	0.398
miR-1	0.85(0.36 ~ 2.93)	1.25(0.73 ~ 1.89)	1.80(0.52 ~ 3.85)	1.34(0.47 ~ 3.02)	3.012	0.390
miR-122	0.28(0.09 ~ 0.56)	0.62(0.15 ~ 1.67)	0.46(0.09 ~ 1.41)	0.33(0.10 ~ 1.32)	3.012	0.390
miR-133a	0.33(0.19 ~ 0.77)	0.58(0.31 ~ 0.92)	0.38(0.23 ~ 0.89)	0.32(0.16 ~ 0.97)	3.797	0.284
miR-145	0.08(0.04 ~ 0.18)	0.07(0.03 ~ 0.18)	0.11(0.04 ~ 0.22)	0.09(0.05 ~ 0.16)	1.308	0.727
miR-214	0.15(0.02 ~ 0.67)	0.11(0.02 ~ 0.34)	0.17(0.04 ~ 0.72)	0.20(0.03 ~ 1.08)	1.436	0.697
miR-21	0.59(0.42 ~ 1.00)	0.82(0.42 ~ 1.55)	0.62(0.44 ~ 0.96)	0.60(0.32 ~ 1.07)	2.457	0.483
miR-25	0.02(0.01 ~ 0.07)	0.03(0.01 ~ 0.11)	0.01(0.01 ~ 0.07)	0.02(0.01 ~ 0.09)	0.950	0.813
miR-20a	0.35(0.14 ~ 0.54)	0.33(0.19 ~ 0.64)	0.34(0.14 ~ 0.58)	0.38(0.18 ~ 0.44)	0.595	0.898
miR-106a	2.69(1.65 ~ 4.31)	2.58(1.92 ~ 4.36)	2.60(1.85 ~ 4.59)	1.92(1.48 ~ 3.81)	1.628	0.653
miR-92a	0.65 ± 0.95	1.29 ± 1.82	1.91 ± 6.21	0.26 ± 0.42	1.544	0.207
miR-130a	59.52(35.26 ~ 88.03)	96.18(38.95 ~ 30.52)	76.90(28.34 ~ 235.57)	46.53(29.24 ~ 97.34)	4.440	0.218
miR-155	159.51(106.22 ~ 337.12)	246.85(154.05 ~ 331.43)	204.36(127.56 ~ 384.01)	158.68(62.25 ~ 314.08)	3.045	0.385
miR-208b	1.05(0.56 ~ 3.90)	0.75(0.59 ~ 1.61)	1.15(0.67 ~ 4.91)	1.30(0.65 ~ 3.38)	1.859	0.602
miR-499	3.10(2.09 ~ 7.26)	2.87(1.89 ~ 5.04)	4.38(2.26 ~ 10.06)	3.75(1.40 ~ 9.29)	2.260	0.520
miR-485-3p	1.56(0.70 ~ 3.38)	1.42(0.84 ~ 2.03)	1.70(0.81 ~ 15.14)	1.87(1.04 ~ 6.59)	1.210	0.751
miR-133b	0.16(0.10 ~ 0.25)	0.18(0.10 ~ 0.22)	0.14(0.09 ~ 0.23)	0.14(0.06 ~ 0.24)	1.251	0.741
miR-221	0.62(0.21 ~ 0.91)	0.43(0.21 ~ 1.12)	0.36(0.21 ~ 0.93)	0.43(0.18 ~ 0.80)	0.648	0.885
miR-208a	1.67(1.12 ~ 3.85)	2.16(1.21 ~ 4.64)	2.25(1.31 ~ 4.39)	2.89(1.69 ~ 4.29)	3.318	0.345
miR-125b	0.17 ± 0.10	0.19 ± 0.10	0.16 ± 0.09	0.12 ± 0.09	2.912	0.037

Data are summarized by either mean ± standard deviation or 50th (25th/75th) percentiles for continuous variables and N1/N2 for binary variables. CHD, coronary heart disease; TCH, total cholesterol; HDL-C, fasting high-density lipoprotein cholesterol; LDL-C, fasting low-density lipoprotein cholesterol; TG, triglyceride; FBG, fasting blood glucose; CR, creatinine. The value of each miRNAs means the relative mount calculated by 2^−Δct^ method.

**Table 3:** Spearman correlations between Gensini score and features of the study population.

Variables	Relationship coefficient	*P* value
Age (years)	0.339	0.000
TCH	0.019	0.841
HDL-C	−0.211	0.021
LDL-C	0.052	0.570
FBG	0.223	0.014
CR	0.276	0.002
miR-433	0.001	0.993
miR-1	0.066	0.469
miR-122	0.071	0.431
miR-133a	0.037	0.681
miR-145	0.043	0.636
miR-214	0.089	0.328
miR-21	−0.057	0.531
miR-25	0.006	0.943
miR-20a	0.007	0.934
miR-106a	−0.079	0.381
miR-92a	−0.151	0.094
miR-130a	−0.066	0.466
miR-155	−0.040	0.660
miR-208b	0.04	0.663
miR-499	0.06	0.507
miR-485-3p	0.084	0.356
miR-133b	−0.083	0.352
miR-221	−0.073	0.422
miR-208a	0.134	0.137
miR-125b	−0.215	0.017

TCH, total cholesterol; HDL-C, fasting high-density lipoprotein cholesterol; LDL-C, fasting low-density lipoprotein cholesterol; TG, triglyceride; FBG, fasting blood glucose; CR, creatinine. The value of each miRNAs means the relative mount calculated by 2^−Δct^ method.

**Table 4:** Receiver operating characteristic curve analyses in subjects with CHD and controls.

Variables	*AUC*(95% *CI*)	*P* value
Age (years)	0.695(0.601 ~ 0.788)	0.000
TCH	0.515(0.411 ~ 0.619)	0.780
HDL-C	0.354(0.255 ~ 0.454)	0.006
LDL-C	0.542(0.438 ~ 0.645)	0.432
FBG	0.647(0.549 ~ 0.745)	0.006
CR	0.622(0.522 ~ 0.723)	0.021
miR-433	0.488(0.386 ~ 0.590)	0.820
miR-1	0.569(0.467 ~ 0.670)	0.189
miR-122	0.547(0.445 ~ 0.649)	0.366
miR-133a	0.527(0.425 ~ 0.630)	0.602
miR-145	0.522(0.419 ~ 0.625)	0.670
miR-214	0.556(0.455 ~ 0.658)	0.281
miR-21	0.461(0.359 ~ 0.562)	0.451
miR-25	0.484(0.381 ~ 0.586)	0.752
miR-20a	0.522(0.419 ~ 0.625)	0.674
miR-106a	0.459(0.357 ~ 0.561)	0.432
miR-92a	0.446(0.344 ~ 0.548)	0.301
miR-130a	0.491(0.388 ~ 0.594)	0.867
miR-155	0.501(0.399 ~ 0.604)	0.978
miR-208b	0.532(0.430 ~ 0.634)	0.540
miR-499	0.546(0.444 ~ 0.674)	0.382
miR-485-3p	0.554(0.452 ~ 0.656)	0.302
miR-133b	0.434(0.333 ~ 0.536)	0.207
miR-221	0.455(0.353 ~ 0.556)	0.384
miR-208a	0.574(0.430 ~ 0.675)	0.159
miR-125b	0.405(0.305 ~ 0.506)	0.070

*CI*, confidence interval; *AUC*, area under the receiver operating characteristic curve; TCH, total cholesterol; HDL-C, fasting high-density lipoprotein cholesterol; LDL-C, fasting low-density lipoprotein cholesterol; TG, triglyceride; FBG, fasting blood glucose; CR, creatinine. The value of each miRNAs means the relative mount calculated by 2^−Δct^ method.

*AUC* < 0.5 indicates the levels in patients with CHD lower than in controls.

*AUC* > 0.5 indicates the levels in patients with CHD higher than in controls.

**Table 5:** The optimal cut-off and the Youden index.

Variables	Cut-off	Sensitivity	Specificity	Youden index
Age	58.500	0.682	0.603	0.285
CR	70.700	0.600	0.679	0.279
FBG	5.590	0.469	0.786	0.255
HDL-C	1.325	0.696	0.594	0.290
miR-125b	0.175	0.534	0.621	0.155

CR, creatinine; FBG, fasting blood glucose; HDL-C, fasting high-density lipoprotein cholesterol; TG, triglyceride. The value of each miRNA-125b means the relative mount calculated by 2^−Δct^ method.

**Table 6:** Synergistic effect of miR-125b and classical risk factors in CHD patients and controls.

Classical risk	miR-125b	CHD	Controls	*OR* (95% *CI*)	*P*value
Age					
0	0	13	18	1	0.002
0	1	8	17	0.652(0.216 ~ 1.962)	0.446
1	0	12	13	1.278(0.443 ~ 3.691)	0.650
1	1	33	10	4.569(1.673 ~ 12.479)	0.003
Sex					
0	0	6	14	1	0.085
0	1	6	7	2.00(0.469 ~ 8.530)	0.349
1	0	19	17	2.608(0.819 ~ 8.309)	0.105
1	1	35	20	4.083(1.355 ~ 12.303)	0.012
CR					
0	0	12	22	1	0.016
0	1	14	16	1.604(0.587 ~ 4.381)	0.357
1	0	13	8	2.979(0.965 ~ 9.196)	0.058
1	1	26	10	4.767(1.731 ~ 13.130)	0.003
FBG					
0	0	13	24	1	0.017
0	1	21	20	1.938(0.779 ~ 4.822)	0.155
1	0	12	6	3.692(1.123 ~ 12.136)	0.031
1	1	18	6	5.538(1.764 ~ 17.391)	0.003
HDL-C					
0	0	10	21	1	0.009
0	1	16	18	1.867(0.680 ~ 5.126)	0.226
1	0	15	9	3.500(1.144 ~ 10.706)	0.028
1	1	23	8	6.037(2.006 ~ 18.173)	0.001

CR, creatinine; FBG, fasting blood glucose; HDL-C, fasting high-density lipoprotein cholesterol.

The value of each miRNA-125b means the relative mount calculated by 2^−Δct^ method.

**Table 7:** The indexes of synergistic effect between miRNAs and classical risk factors.

Variables	*SI*	*SIM*	*RERI*	*AP*
Age-miR-125b	−50.99	5.48	3.64	0.80
Sex-miR-125b	1.18	0.78	0.48	0.12
CR-miR-125b	1.46	1.00	1.18	0.25
FBG-miR-125b	1.25	0.77	0.91	0.16
HDL-miR-125b	1.50	0.92	1.67	0.28

*SI*, synergy measures in additive models; *SIM*, synergy measures in multiplicative models; *RERI*, relative excess risk due to interaction; *AP*, proportion of disease attributable to interaction; CR, creatinine; FBG, fasting blood glucose; HDL-C, fasting high-density lipoprotein cholesterol.
